# Hitchhiking to the abyss

**DOI:** 10.1002/ece3.10126

**Published:** 2023-05-28

**Authors:** Jorge Fontes, Gloria Castellano‐González, Bruno C. L. Macena, Pedro Afonso

**Affiliations:** ^1^ Ocean Sciences Institute – Okeanos University of the Azores Horta Portugal; ^2^ Institute of Marine Research – IMAR University of the Azores Horta Portugal

**Keywords:** common remora, deep diving, hitchhikers, pilot fish

## Abstract

We investigated, for the first time, the hitchhiker‐host fidelity of deep‐diving whale sharks and Chilean devil rays. We found that two of the most ubiquitous oceanic hitchhikers, the common remora and the pilot fish, are able to follow their hosts to bathypelagic depths, where they are exposed to extreme gradients of light, dissolved oxygen, temperature, and pressure. We documented a deep dive of a large whale shark hosting remoras and pilot fish. Common remora was observed at the deepest section of the dive, at 1460 m, where the water temperature was 3.6°C. A pilot fish was recorded at 900 m, during the ascent phase, with the water temperature of 7.5°C. Although the adaptations that allow these hitchhikers to mitigate the impacts of such extreme environmental conditions remain unknown, we discuss these findings in the framework of the ecophysiology of deep diving and the hitchhiker‐host fidelity.

## Graphical abstract

We found that common remora and the pilot fish are able to follow their hosts to bathypelagic depths, where they are exposed to extreme gradients of light, dissolved oxygen, temperature, and pressure. Although the adaptations that allow these hitchhikers to mitigate the impacts of such extreme environmental conditions remain unknown, we discuss these findings in the framework of the ecophysiology of deep diving and the hitchhiker‐host fidelity.

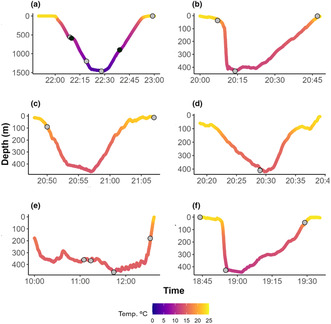

## 1 INTRODUCTION

**FIGURE 1:**
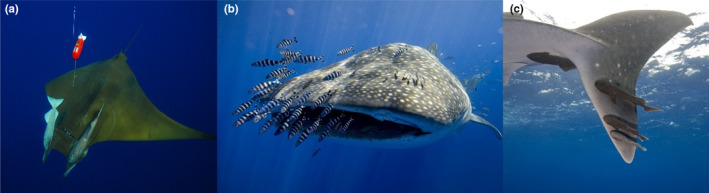
Common remora—*Remora remora* (a, c) and pilot fish—*Naucrates ductor* (b), associated with Chilean devil ray—*Mobula tarapacana* (a) and whale shark—*Rhincodon typus* (b, c). Note that panel a also represents the noninvasive harness with towed PILOT tag (Fontes et al., 2022). Photo credits: J. Fontes Okeanos‐*Imag*DOP©.

Remoras (Echeneidae) and pilot fish (Carangidae) belong to the order of Carangiformes (Nelson et al., 2016) and are among the most common hitchhikers associated with marine megafauna, including sharks and mobula rays (Figure 1). Remoras typically attach to their hosts using the highly specialized suction disc. Some remoras are reef generalists that have no clear host preference, whereas others, such as the common remora (*Remora remora*) are pelagic specialists, which can be found associated with 30 different hosts, but more frequently with sharks (O'Toole, 2002) and mobulas (Becerril‐García et al., 2019; Nicholson‐Jack et al., 2021). Pilot fishes (*Naucrates ductor*) are normally found actively swimming closely associated with pelagic megafauna or sometimes associated with floating objects (J. Fontes & B. Macena, personal observation; Pipitone et al., 2000). Both the pilot fish and six species of remora have been reported in the Azores archipelago (Santos et al., 1997), however, the most frequent is the common remora (*R. remora*). The common remora is often found together with pilot fishes, mainly associated with whale sharks (*Rynchodon typus*), blue sharks, and Chilean devil rays (*Mobula tarapacana*, hereafter devil ray).

Although circumglobally present, we know surprisingly little about the natural history and ecology of the pilot fish, including direct observations of their vertical distribution range. The ecology and anatomy of remoras and the remora–host relationship have received far more attention, possibly due to the interest in understanding the impact of hitchhikers on iconic marine megafauna hosts (Xu et al., 2021). In addition, the unique properties of remora's attachment mechanism have fuelled the research of bioinspired technological solutions (e.g., Lee et al., 2019; Su et al., 2020; Wang et al., 2022). Yet, our understanding of the evolutionary drivers of such an exceptional and complex structure remains limited since all known remora hosts evolved before remoras themselves (Gamel et al., 2019).

Hitchhiking behavior is central in the ecology of remoras and pilot fish as well as their hosts. The hosts potentially provide protection from predators, access to food, and allow energy saving by reducing the cost of transport, either by attaching to the host (remoras; Fuller & Parsons, 2019) or by riding the wake of the swimming host (remoras and pilot, Fontes, personal observation). Yet, the nature of the relationship between hitchhikers and hosts remains a matter of debate. In some cases, both may benefit from the association (mutualism), for example, when the hitchhikers reduce the cost of transport and receive protection from predators and food while removing parasites from the host, there are other situations where the host may receive no net benefit (commensalism; Mathis & Bronstein, 2020). There are, however, situations where remoras can produce parasitic drag and skin abrasion on the hosts, suggesting that the relationship could also be antagonistic (Brunnschweiler et al., 2020). To what extent is this relationship beneficial for the host, neutral or has a negative impact, might be specific for each hitchhiker–host pair and the context of the association (Mathis & Bronstein, 2020).

Despite the significance of the hitchhiker–host relationship, we remain unaware regarding host fidelity for remoras and pilot fish because investigating hitchhiker–host ecology is quite challenging in the wild, especially when the hosts (devil rays and whale shark) are capable of diving into bathyal depths of nearly 2000 m (Thorrold et al., 2014; Tyminski et al., 2015). Notwithstanding the host's deep‐diving behavior, the maximum confirmed depth for the common remoras is 140 m when they were observed from a submersible while their hosts, the oceanic mantas (*Mobula birostris*), were feeding (Stewart et al., 2016). The maximum depth for the pilot fish is unknown, although Fricke et al. (2011) reported a 0–300 m vertical range. Yet, as far as we know, there are no direct observations available to confirm this range. Regardless, the current notion of maximum depth range for these hitchhikers is difficult to conciliate with the deep‐diving habits of some of their common hosts, and we remain oblivious to the hitchhiker–host fidelity during deep diving and its ecological and physiological implications. It is generally accepted that one of the potential drivers of deep diving may be related to parasite offload (Braun et al., 2022). Thus, considering that remoras can increase the cost of transport and cause skin abrasion on their hosts (Brunnschweiler et al., 2020; G. Castellano‐González, T. Bartolomeu, A. Passos, B. C. L. Macena, P. Afonso, & J. Fontes, unpublished), deep diving could serve to dislodge and control the hitchhiker load and manage the parasitic drag.

Here, we investigated the hitchhiker–host fidelity during deep dives by analyzing the videos from animal‐borne multisensor cameras tags (Fontes et al., 2022), deployed on free swimming adult whale sharks and Chilean devil rays, in the Azores archipelago, Portugal.

## 2 METHODS

We analyzed 42 vertical profiles from multisensor camera tag deployments available on our database of animal‐borne camera tags, deployed on free‐swimming whale sharks and devil rays from the Azores region (NE Atlantic), where video and depth data were collected simultaneously, using noninvasive i‐Pilot towed tags (for details, see Fontes et al., 2018, 2022). In short, the i‐Pilot tag is torpedo‐shaped, composed of two symmetrical floats (200 bar rated syntactic foam), held together by bolts and pins, to secure the electronic components and structural elements, stabilized by vertical and horizontal fins. At the core is the titanium housing with a viewport, containing the camera (Sony IMX219, 1920 × 1080 @ 30 fps) and all the sensors and batteries, connected to external red LEDs (Fontes et al., 2022). Satellite and VHF transmitters are located in the dorsal section of the tag as well as a magnetic paddle wheel to measure the speed. The i‐Pilot camera tags (Fontes et al., 2022) were attached to whale sharks and devil rays using self‐releasing fin clamps and harnesses, respectively (Fontes et al., 2018, 2022). Briefly, an off‐the‐shelf nylon clamp was fitted with a galvanic time release (GTR) to release the clamp and tag after 3–24 h. The i‐Pilot tag was connected to the camp with a 150‐cm‐long monofilament line. A free diver attached the tag by securing the clamp on the anterior edge of the pectoral fin of the whale shark. The devil ray harness was composed of an elastic rope (6 mm thickness and 140 cm long), threaded through a stainless‐steel ring, and attached to either end of a GTR forming a loop. A 100‐cm‐long piece of monofilament line was then tied to the metallic ring and secured to the Pilot tag. Harnesses were deployed on free‐swimming mobulas by free divers, by stretching the loop, with open arms in a U shape, to fit the harness around the head and rest it on the mobulids “shoulders.”

We inspected the footage from the deep (meso and bathypelagic) dive profiles available and recorded the presence of either common remora or pilot fish and the corresponding time, depth, and temperature. We used the MPC‐HC (https://mpc‐hc.org) video player for visualization and Igor Pro‐ver. 8.0 (Wavemetrics, Inc. Lake Oswego, USA), with the package Ethnographer to analyze the depth and water temperature profiles.

## 3 RESULTS AND DISCUSSION

**FIGURE 2:**
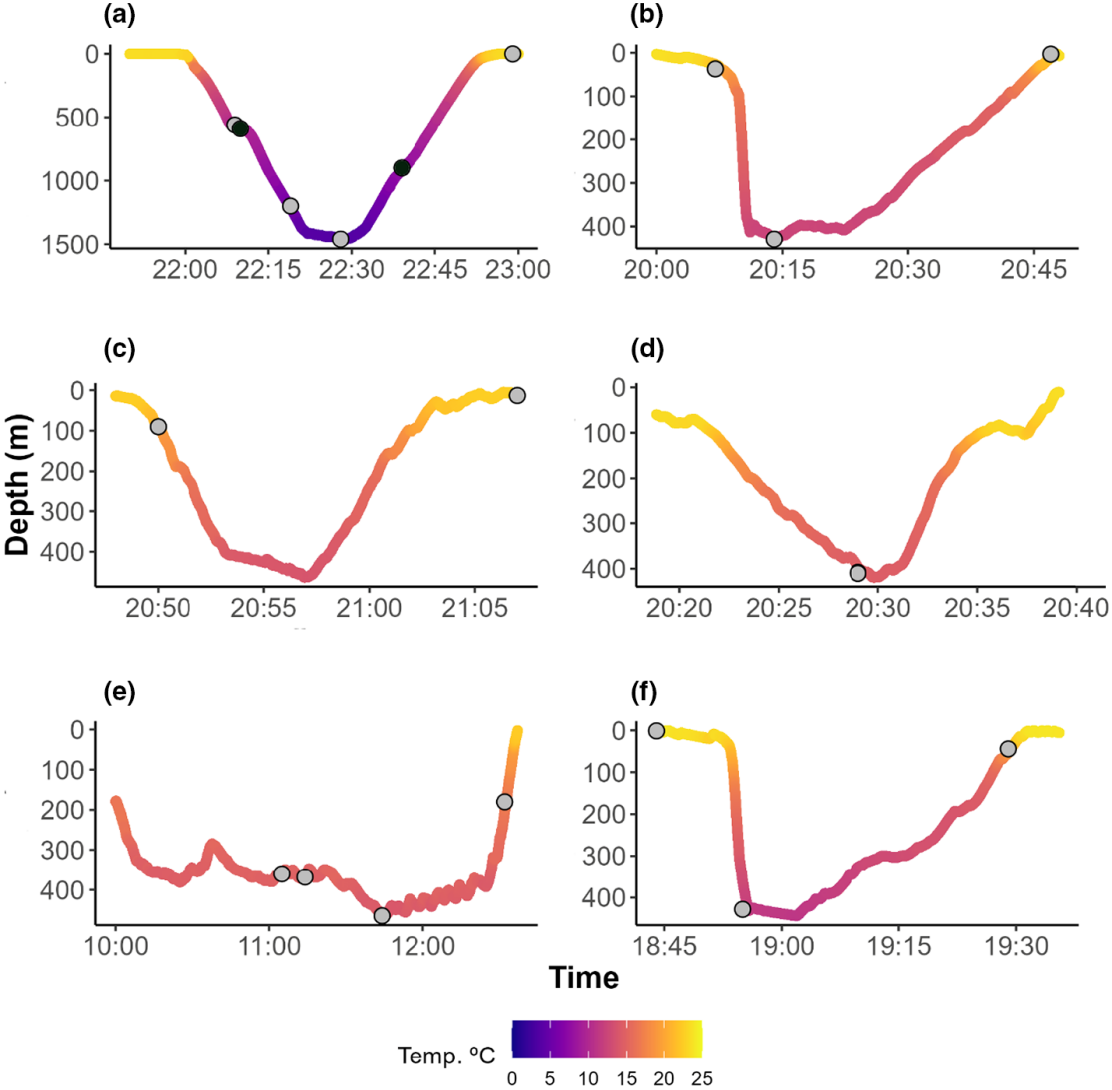
Depth and temperature profiles of selected deep dives (>400 m) of the tagged whale shark—*Rhincodon typus* (a) and Chilean devil rays—*Mobula tarapacana* (b–f). Gray and black circles represent common remora—*Remora remora* and pilot fish—*Naucrates ductor* recorded on video, respectively. Note the differences in the range of depth.

**FIGURE 3:**
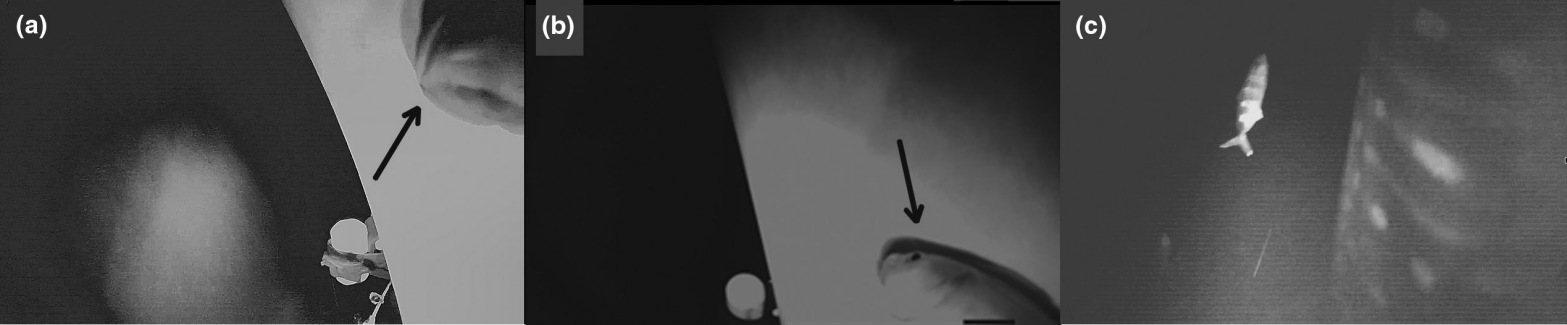
Video frame from the tagged whale shark—*Rhincodon typus*, showing (a) the deepest record of *Remora remora*, showing a ventral view of the head of the attached remora signaled with the black arrow, (b) lateral anterior view of attached remora signaled with the black arrow, and (c) lateral view of a pilot fish, *Naucrates ductor*, swimming in front of the camera. Note the seafloor reflects the camera tag lights in the center of panel a.

The deepest dive profile is available in our multispecies camera telemetry database, where hitchhikers were visiblly performed by an 800 cm (estimated total length) female whale shark, in August 2020, off Santa Maria island (37.0254°N; 25.18735°W). The whale shark started the dive at 10:00 p.m., and descended for 28 min, at an average descent rate of 0.86 ms^−1^, with the water temperature ranging between 23.5 and 3.6°C, from the surface to the deepest section at 1460 m, where the seafloor was visible for a few seconds. After 60 s at the maximum depth, the whale shark started ascending, at an average ascent rate of 0.97 ms^−1^, reaching the surface 25 min later, completing a typical V‐shape dive (Figure 2a). The deepest common remora was observed at 1460 m, with a water temperature of 3.6°C, when it was attached to the ventral side of the left pectoral fin (Figure 3a and Videos S1 and S2). The deepest pilot fish associated with this host was recorded at 900 m, during the ascent phase, when the water temperature was 7.5°C (Figures 2a and 3b, and Video S3). Although the pilot fish likely followed its whale shark host over the entire dive, down to almost 1500 m and the water temperature below 4°C, the video footage can only confirm its presence halfway through the descent, below 500 m and at about 900 m, when completing the first third of the ascent stage.

We also analyzed five mesopelagic dives of camera‐tagged devil rays where common remora were visible deeper than 400 m (Figure 2b–f). Remoras and devil rays were exposed to temperatures ranging from 23.9 to 7.2°C.

These new observations significantly expand our understanding regarding the tolerance of two of the most common megafauna hitchhikers to extreme environmental gradients of pressure, temperature, and dissolved oxygen, suggesting that they may have unknown and extraordinary anatomical and physiological adaptations to mitigate the impact of deep diving.

Deep‐diving animals (including some hosts) have developed specific adaptations to overcome these challenges and mitigate the impacts of (i) extremely cold temperatures and low oxygen concentrations, which can hinder the proper functioning of organs; (ii) low light levels, which can preclude visual targeting of prey, conspecifics, other predators, and even the host; and (iii) high pressure, which can potentially cause decompression sickness and influence enzyme performance (Braun et al., 2022).

Several marine animals rely on some form of endothermy to overcome or attenuate cold conditions at a depth that reduces metabolic rates. Regional endothermy is present in some elasmobranchs, including the Chilean devil ray, in the form of vascularized systems acting as countercurrent heat exchangers (retia mirabilia), which conserve body heat by transferring it among neighboring blood vessels flowing in opposite directions (Alexander, 1996; Schweitzer & Notarbartolo‐Di‐Sciara, 1986). Other species regulate their body temperature by returning repeatedly to shallower water to rewarm after deep dives, as well as physiological thermoregulation, in which they alter their blood circulatory routes to minimize heat loss while diving into cold water and maximize heat gain while ascending into warm water (e.g., common mola, Nakamura et al., 2015). Large animals, such as the whale shark, rely on the fact that they have high thermal inertia and a low surface area to volume ratio, which helps maintain body temperature. In fact, the whole‐body heat‐transfer coefficients of whale sharks are the lowest of any fish species measured to date (Nakamura et al., 2020). In contrast, it is unknown how remoras and pilot fishes are able to mitigate the effects of extremely cold temperatures when they follow their hosts to the bathypelagic. These small‐bodied fishes cannot rely on thermal inertia to mitigate the drop in ambient water temperature at depth and no thermoregulation nor the presence of anatomical adaptations for thermoregulation has been reported. Remoras and pilot fish possibly combine physiological adaptations and/or behavioral strategies to alleviate the effects of the extreme cold. We cannot rule out the possibility that these hitchhikers may possess thermoregulatory capabilities until detailed physiological and anatomical studies are available.

Some elasmobranchs can maximize oxygen uptake from hypoxic waters at depth by increasing myoglobin concentrations in locomotory muscles that aid in the diffusion and storage of oxygen, for example, thresher and mackerel sharks (Bernal et al., 2003). While bony fishes like the bigeye tuna can increase the oxygen affinities in their blood to enhance available oxygen (Lowe et al., 2000), others have increased their gill surface area to enhance hypoxia tolerance by augmenting oxygen uptake (e.g., tunas, billfishes; Wegner et al., 2010). Similar adaptations are not known for either the hosts or the hitchhikers studied here.

The bathypelagic is also characterized by extremely low light. As a result, deep‐diving sharks and tunas developed a specialized reflective tissue in their eyes, the *tapetum lucidum*, which increases photosensitivity (Collin, 2018), allowing for greater raw and residual visual acuity (correcting for relative eye investment) in deep‐diving elasmobranchs than in epipelagic and deep‐sea species. The presence of visual adaptations to enhance visual acuity in hitchhikers is unknown. Although acute vision may not be important for an attached hitchhiker, such as the common remora, pilot fish may have to combine vision with other senses to follow and maintain optimal positioning relative to their deep‐diving hosts.

The immense pressures experienced at bathypelagic depths, along with rapid changes during descent and ascent, pose an important challenge to both hosts and hitchhikers. While elasmobranchs lack a gas bladder, many deep‐diving teleost fish (e.g., tunas) have lost this organ to allow rapid vertical movements and swim at high speed (McCune & Carlson, 2004). For the Echneidae family, lacking a gas bladder allows them to attach to hosts that move rapidly up and down in the water column (e.g., sharks, turtles, whales; McCune & Carlson, 2004). Although the buoyancy control mechanisms of the pilot fishes are unknown, other Carangidae developed alternative solutions to mitigate the risk of gas bladder rupture during rapid ascents. For example, several jacks (*Seriola hippos*, *S. lalandi*, *S. dumerili*, and *Pseudocaranx georgianus*) retained the gas bladder to maintain hydrostatic stability but developed a specialized venting structure to vent this organ and alleviate pressure buildup during fast ascents (Hughes et al., 2016). Detailed analysis of pilot fish anatomy and simple air injection test, as suggested by Hughes et al. (2016), should help us understand how pilot fish control flotation and mitigate the impact of extreme pressure variations.

This is the first time that hitchhikers were observed to follow their hosts to the bathypelagic, suggesting that deep diving may not be sufficient to dislodge large remora, despite the parasitic drag and increased cost of transport resulting from their attachment (Flammang et al., 2020). For example, a single 40 cm remora can increase the cost of transport of a 300 cm devil ray host by 2%–4%. This penalty will increase linearly with additional remoras attached, which normally range between one and three (Castellano et al., submitted). In contrast, the parasitic drag added by a single remora on an 800 cm whale shark is negligible. However, whale sharks can sometimes host as many as two dozen large remoras (J. Fontes & B. Macena, unpublished data), which may have a relevant impact on the cost of transport over the long term.

In summary, we found that two of the most ubiquitous oceanic hitchhikers can follow their deep‐diving hosts to bathypelagic depths, where they are exposed to extreme environmental gradients of light, dissolved oxygen, temperature, and pressure. Only a few large‐bodied teleost fishes, such as tunas (e.g., bluefin, etc.), were previously known to tolerate such gradients. Yet, the adaptations that allow the common remora and the pilot fish to mitigate the impacts of extreme environmental conditions remain unknown. Further research and experiments are needed to understand the anatomical and physiological mechanisms involved as well as the cost of deep diving for these hitchhikers.

## AUTHOR CONTRIBUTIONS

**Jorge Fontes:** Conceptualization (equal); funding acquisition (equal); investigation (equal); methodology (equal); project administration (equal); resources (equal); supervision (equal); writing – original draft (equal). **Gloria Castellano‐González:** Conceptualization (supporting); data curation (supporting); formal analysis (equal); investigation (supporting); writing – review and editing (equal). **Bruno C. L. Macena:** Conceptualization (equal); data curation (equal); formal analysis (supporting); funding acquisition (supporting); investigation (equal); methodology (equal); supervision (supporting); writing – review and editing (lead). **Pedro Afonso:** Conceptualization (supporting); funding acquisition (equal); investigation (supporting); project administration (supporting); resources (supporting); supervision (equal); writing – review and editing (supporting).

## CONFLICT OF INTEREST STATEMENT

The authors declare that they have no conflict of interest.

## Supporting information


Video S1

Video S2

Video S3

Supplementary Videos Captions

## Data Availability

Selected deep sections of the animal‐borne video with visible hitchhikers, common remoras, and pilot fish associated with whale sharks are available at https://figshare.com/articles/media/Hitchhikers_deepdive/22249528.
