# Supplementary material for: Hitchhiking to the abyss
Source: Ecol Evol. 2023 May 28;13(5):e10126. doi: 10.1002/ece3.10126 (PMC10225908; doi:10.1002/ece3.10126)
Supplement: Supplementary file 4 — Supplementary Videos Captions [file ECE3-13-e10126-s003.docx]

**Video S1.** Video of *Remora remora* attached to *Rhincodon typus* at the deepest section of a 1460m dive.

**Video S2.** Video of *Remora remora* at 940m attached to *Rhincodon typus* during descent phase of deep a dive.

**Video S3.** Video of *Naucrates ductor* attached to *Rhincodon typus* recorded at 900 m, during the ascent phase of a deep dive.
